# The Comparative Assessment of Wellens’ Syndrome With Proximal Left Anterior Descending Artery (LAD) Stenosis Versus Right Coronary Artery (RCA) or Circumflex Coronary Artery Stenosis and Its Prevalence: A Systematic Review

**DOI:** 10.7759/cureus.37991

**Published:** 2023-04-22

**Authors:** Mukosolu F Obi, Vikhyath Namireddy, Chelsea Noel, Aidan O’Brien, Manjari Sharma, Ariel Frederick, Blossom De Gale, Manveer Ubhi, Ryan Cho

**Affiliations:** 1 Internal Medicine, Wyckoff Heights Medical Center, Brooklyn, USA; 2 Medicine, St. George’s University School of Medicine, True Blue, GRD

**Keywords:** wellens’ syndrome, left anterior descending artery (lad), left circumflex artery, right coronary artery, t wave pattern, proximal stenosis

## Abstract

Wellens’ syndrome is well-known for its critical stenosis of the proximal left anterior descending artery (LAD) with characteristic electrocardiographic findings of biphasic or deeply inverted T waves in V2-V6 under specific diagnostic criteria. Although the syndrome is known as a high-grade LAD lesion, its sequence of events can also be seen with the right coronary artery (RCA) and the left circumflex artery (LCX). This systemic review attempts to expand on these findings while analyzing the prevalence of Wellens’ syndrome with the RCA and/or the circumflex artery. This study also comparatively indicated that Wellens’ syndrome is seen in RCA and circumflex artery stenoses when present; the indication of the same medical management is warranted for effective treatment and survival. We extracted and analyzed 24 case reports each with an atypical presentation of acute coronary syndrome (ACS) and specific Wellens’ syndrome pattern of electrocardiogram (ECG) presentation with critical stenosis in the LAD, RCA, and left circumflex artery. The risk of bias assessment was undertaken using internal risk analysis by utilizing medical libraries and certain search phrases to find research articles with the involvement of the LAD as opposed to the RCA and LCX in Wellens’ syndrome. Based on the number of respective primary research articles found, a bias calculation was done on the reported respective coronary artery involvement.

The finding of our systemic review confirms that Wellens’ syndrome is a precordial lead disease with T wave abnormalities that present with critical stenosis of not only the LAD but also the RCA and circumflex artery.

The result of our systemic review affirmed that although most Wellens’ syndrome cases reported involve the stenosis of the LAD, the critical occlusion of the RCA and/or the circumflex artery was found with Wellens’ syndrome pattern of ECG presentation, meaning that the sequence of events is not limited to the proximal LAD.

## Introduction and background

Wellens’ syndrome is a clinical syndrome with a specific pattern of electrocardiogram (ECG) characterized by biphasic or deeply inverted T waves seen in precordial leads in V1-V6, which can indicate critical blockage or stenosis in one or more coronary arteries. Wellens’ syndrome was first discovered by de Zwaan et al. in 1982 while evaluating patients with unstable angina during a pain-free period. Since then, Wellens’ syndrome gained recognition as a diagnostic tool to identify high-risk patients with acute coronary syndrome (ACS) [1]. Wellens’ syndrome is traditionally seen with proximal left anterior descending artery (LAD) stenosis with either biphasic (25% of cases: type A) or deeply inverted (75%: type B) T waves in V2-V3. These ECG findings are often missed during triage on patients with high-risk factors for ACS. However, recent studies have suggested that the syndrome is not limited to proximal LAD but is also associated with critical stenosis in the right coronary artery (RCA) and/or the left circumflex artery (LCX).

The phenomenon of coronary artery stenosis, reperfusion, and restenosis is a characteristic pathophysiology of Wellens’ syndrome, and as a result, most of the presentations are during a pain-free period. The criteria for considering the diagnosis of Wellens’ syndrome include the following: a prior occurrence of discomfort or pain in the chest area; chest discomfort or pain that does not show abnormalities on an ECG, which can sometimes mask the presence of underlying heart disease (pseudo normalization); a slight increase in the levels of cardiac enzymes in the blood, which may suggest cardiac damage but are not significantly elevated; the absence of abnormal Q waves or R wave progression in the precordial leads of the ECG; a slight elevation of the ST segment in leads V2-V3 without associated pain; and symmetrical deep inversion of T waves or T waves with two positive deflections knowns as biphasic T waves in leads V2-V5 without associated pain [2]. These criteria when recognized require immediate medical attention, although Wellens’ syndrome can be seen in normal coronary arteries following an episode of vasospasm as in patients with cocaine-induced vasospasm, invasive medical intervention such as coronary angiogram is required to rule out critical stenosis and if present will require percutaneous coronary intervention (PCI) or coronary artery bypass grafting (CABG) as medical therapy is inadequate. Our systemic review aims to expand and explore other studies with cases indicating Wellens’ syndrome with coronary stenosis other than the LAD but the RCA and/or the LCX.

## Review

Methodology

We utilized the population, intervention, control, and outcomes (PICO) model and the Preferred Reporting Items for Systematic Reviews and Meta-Analyses (PRISMA) framework to perform our systematic review.

Eligibility Parameters

In our systematic review, we included case reports that described patients over the age of 18 who were diagnosed with Wellens’ syndrome upon the evaluation of their electrocardiogram (ECG) findings and whose cardiac catheterization findings revealed left anterior descending artery (LAD) occlusion, left circumflex artery (LCX) occlusion, right coronary artery (RCA) occlusion, or multivessel occlusions. To assess whether Wellens’ syndrome can indicate the occlusion of other coronary vessels besides the proximal LAD, we used the following inclusion criteria: ECG pattern of deeply inverted or biphasic T waves V1-V6 present in a pain-free state, normal or slightly elevated serum cardiac markers, no precordial R wave progression, no precordial Q waves, isoelectric or minimally elevated ST segment (<1 mm), and cardiac catheterization revealing the occlusion of the LAD only, LCX only, RCA only, or multivessel occlusion.

Electronic Searches

To acquire and explore the articles of interest in this systemic review, three independent authors searched across the following databases: PubMed, Google Scholar, ScienceDirect, Cochrane, Journal of the American Medical Association, New England Journal of Medicine, and National Library of Medicine. The articles found were published between 2013 and 2023. The search for our requisite articles was conducted between March 6 and March 9, 2023. The exploration conducted had the following keywords: “Wellens,” “Wellens syndrome,” “Wellens Left Circumflex,” “Wellens LCX,” “Wellens in Right Coronary,” “Wellens in LAD,” “LAD,” “left anterior descending artery,” “inverted t-waves,” and “biphasic t-waves.” Our search employed these terms with Boolean operators to derive our requisite articles. We restricted the search to the English language and screened abstracts before ensuring full-text articles matched our criteria for eligibility.

Data Collection and Assessment

Three dedicated authors collected data based on the eligibility parameters. Each author collected data that illustrated the stenosis of the RCA, circumflex artery, and LAD. Each author analyzed the patient presentation, laboratory testing and imaging studies, and the outcome of early intervention for the respective stenosed artery.

The data collected was inclusive of numerous case studies and a prospective analysis. We excluded studies that were bereft of positive ECG findings, systematic reviews, case reports, and cardiac interventions. The authors discussed their opinions regarding data collection and mutually resolved their differences. A concerted consensus was developed for the systematic review of the case studies. The authors analyzed the incidence of Wellens’ syndrome with the involvement of the RCA and circumflex artery, atypical presentations of patients with Wellens’ syndrome, and the outcome of early intervention. The findings were recorded electronically, and the data was transferred to the study table.

Risk of Bias Assessment

**Figure 1:**
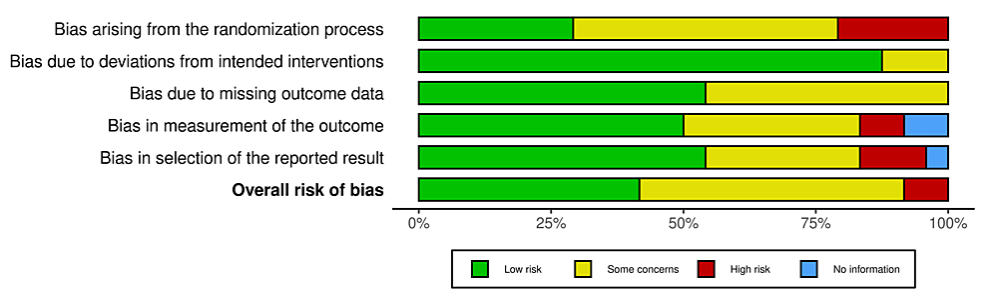
Risk of bias assessment of reported cases

**Figure 2:**
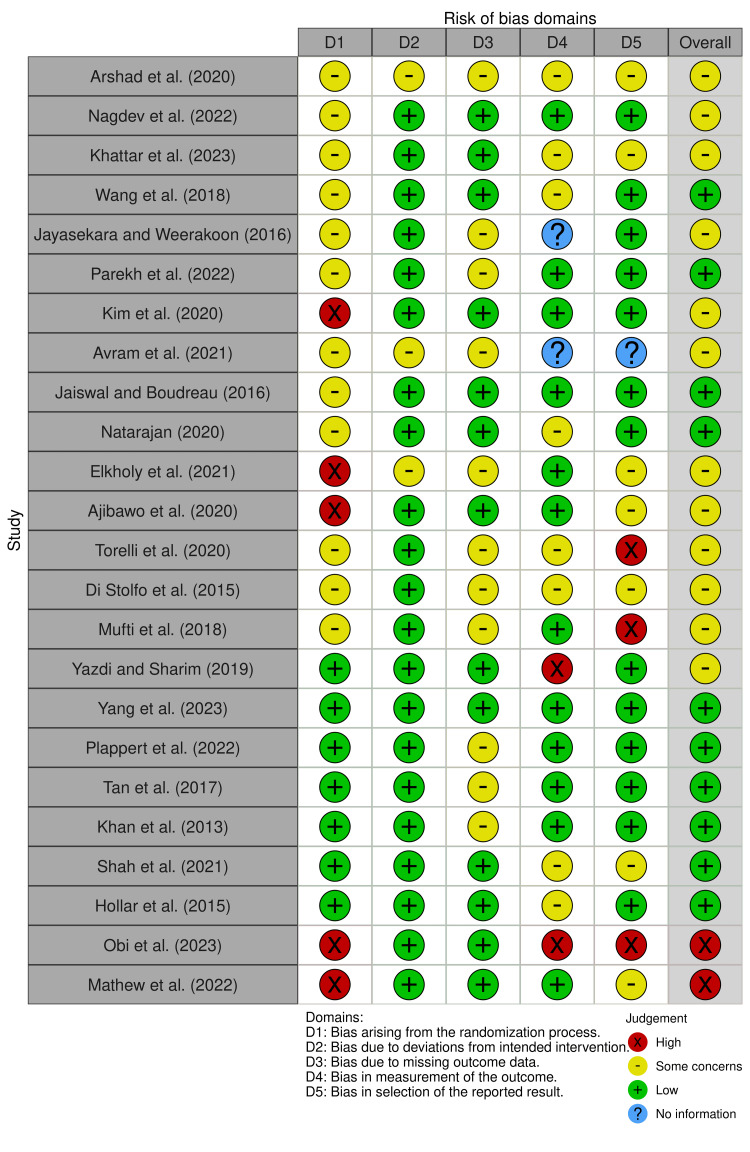
Reported cases: risk of bias assessment Arshad et al. (2020) [3], Nagdev et al. (2022) [4], Khattar et al. (2023) [5], Wang et al. (2018) [6], Jayasekara and Weerakoon (2016) [7], Parekh et al. (2022) [8], Kim et al. (2020) [9], Avram et al. (2021) [10], Jaiswal and Boudreau (2016) [11], Natarajan (2020) [12], Elkholy et al. (2021) [13], Ajibawo et al. (2020) [14], Torelli et al. (2020) [15], Di Stolfo et al. (2015) [16], Mufti et al. (2018) [17], Yazdi and Sharim (2019) [18], Yang et al. (2023) [19], Plappert et al. (2022) [20], Tan et al. (2017) [21], Khan et al. (2013) [22], Shah et al. (2021) [23], Hollar et al. (2015) [24], Obi et al. (2023) [25], Mathew et al. [2]

Using the Cochrane risk-of-bias tool (RoB2), two authors independently scrutinized the randomness of sequence processes (selection bias), adherence to intended interventions, incompleteness of outcome data, accuracy of outcome measurement, and selection of reported outcomes. The evaluation of selection bias in the random sequence processes was based on the articles chosen according to the degree of stenosis in the coronary arteries of the patients, with the LAD being deemed a low-bias artery, mixed stenosis indicating some bias, and articles highlighting LCX or RCA stenosis being subject to high bias. The primary interventions examined were PCI or CABG, with articles focusing on these being assigned low bias, mixed management indicating some bias, and primarily medical management being subject to high bias. The completeness of outcome data was evaluated based on the criteria for a classic Wellens’ syndrome presentation. The measurement of the outcome was determined by the risk of coronary artery stenosis in each patient. Lastly, reporting bias was assessed based on the reasonableness of reporting these cases as Wellens’ syndrome considering the patient’s clinical presentation (Figures 1-2).

Outcome Measurement

A group of three authors carefully evaluated a selection of articles that fulfilled their inclusion criteria, focusing on the presence of Wellens’ syndrome criteria and coronary arteries other than the well-known LAD, such as the LCX and RCA. Although the LAD is typically associated with Wellens’ syndrome, the authors also considered and was able to note and record stenosis in the LCX and RCA, or a combination of stenosis, to meet their Wellens’ syndrome criteria.

Missing Data

The authors worked together to ensure that only relevant articles were included in their systematic review, excluding those that were limited to abstracts or fell outside of their search criteria. Additionally, reports and studies that did not meet the criteria for Wellens’ syndrome or lacked necessary information on stenosed arteries were also removed from consideration.

Review

**Figure 3:**
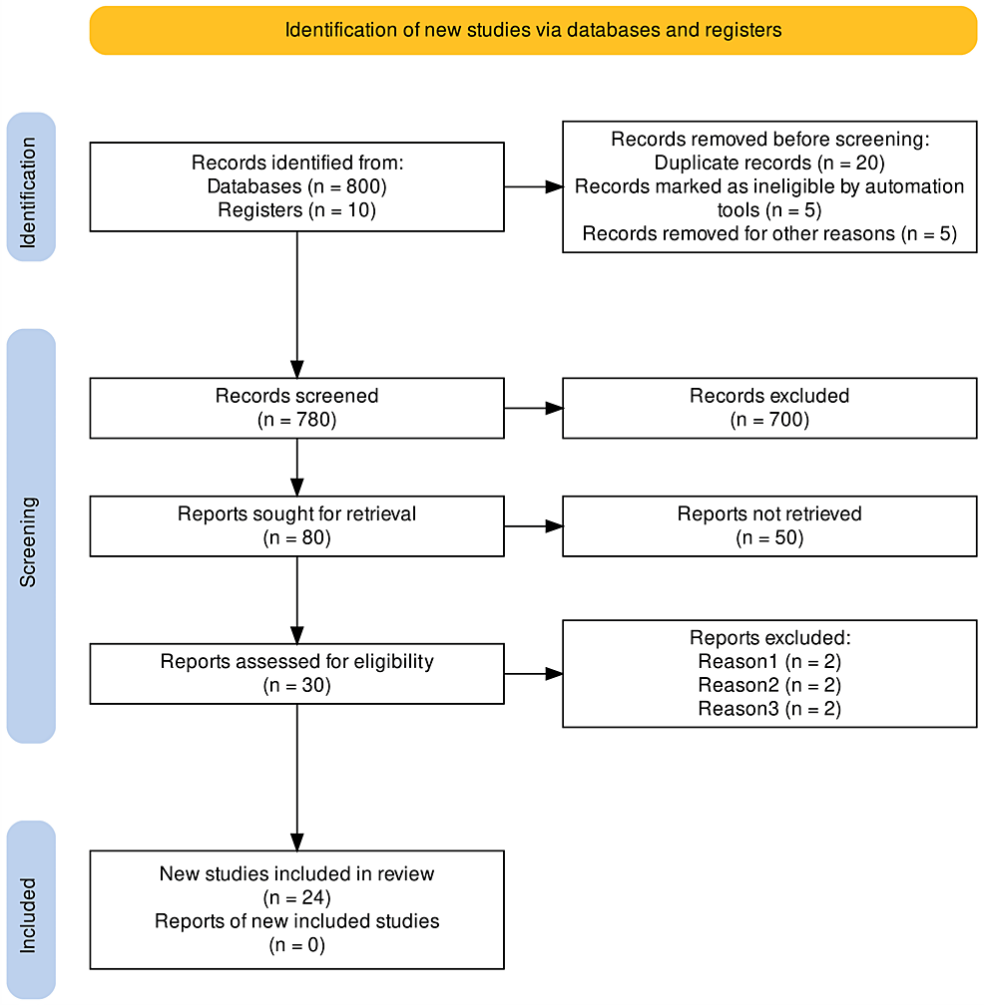
PRISMA flow diagram of the study screening process PRISMA: Preferred Reporting Items for Systematic Reviews and Meta-Analyses

We extracted and analyzed 24 out of 800 with features pertaining to Wellens’ syndrome (Figure 3) [26]. These primary research papers met the inclusion protocols we set, which included the diagnostic criteria known for Wellens’ syndrome and the targeted coronary arteries of interest.

These studies revealed that Wellens’ syndrome not only is limited to the proximal LAD but also can be seen in the RCA and LCX or both. It is a multivessel coronary disease. These primary research cases affirmed the presence of Wellens’ syndrome following the diagnostic criteria in the RCA and LCX. A total of 12 cases were found to have critical stenosis with RCA involvement, 12 with critical stenosis involving LCX, and 20 cases with critical stenosis involving the proximal LAD. Although, in most of the cases, the LAD is recognized as the most culprit coronary artery involved in the multivessel occlusion, some cases reported isolated critical stenosis in the LCX and RCA. Based on the analysis and comparison of cases/study design, Wellens’ syndrome often involves multivessel disease, and in each case, the patient presents with atypical presentation or intermittent chronic anginal symptoms. One study presented Wellens’ syndrome with coronary artery angiogram (CAG) result indicating RCA stenosis with ostial 50%-60% focal stenosis, 70%-80% proximal RCA stenosis, and 80% distal stenosis at the bifurcation of the right posterior descending artery and right posterior left ventricular artery [2]. Another study releveled Wellens’ syndrome with CAG indicating 85% stenosis of the proximal circumflex artery and mild atherosclerotic disease of the LAD [14]. One study affirmed multivessel critical stenosis: 100% stenosis in the proximal RCA and LCX, 90% stenosis in the middle LAD, and 95% stenosis in distal LAD [25]. The overall findings claimed that although Wellens’ syndrome has high prevalence with LAD stenosis, it is often noted to have multivessel disease, with the involvement of the coronary arteries not limited to the LAD but also involves RCA or LCX or both. Table 1 presents the primary research studies extracted.

**Table 1:** Baseline analysis of included studies ECG, electrocardiogram; LAD, left anterior descending artery; LCX, left circumflex artery; RCA, right coronary artery; PCI, percutaneous coronary intervention; CABG, coronary artery bypass grafting; PTSD, post-traumatic stress disorder; ADHD, attention-deficit/hyperactivity disorder; BPD, borderline personality disorder; NSTEMI, non-ST elevation myocardial infarction; CAD, coronary artery disease; T2DM, type 2 diabetes mellitus; GERD, gastroesophageal reflux disease

Author	Case Presentation	Study Design	Coronary Arteries Involved	Intervention
Arshad et al. (2020) [3]	The authors utilized the MUSE ECG database of Montefiore Medical Center to identify ECGs from 2012 to 2019 exhibiting a Wellens’ syndrome pattern. The results of the study showed that patients with a Wellens’ syndrome pattern may have critical lesions at a variety of LAD sites and in multivessels	Systemic review	LAD, LCX, and RCA	-
Ajibawo et al. (2020) [14]	The authors indicated a typical presentation of Wellens’ syndrome. The patient presented with epigastric pain and syncope	Case report	LCX	PCI
Avram et al. (2021) [10]	The authors performed an analysis of patients with acute coronary syndrome (ACS) and Wellens’ sign who underwent coronary angiography between January 2018 and December 2019. The results of the study showed that the LAD was the principal vessel involved for most patients. Significant LCX and RCA involvement was also seen	Prospective single-center cohort study	LAD, LCX, and RCA	PCI and CABG
Nagdev et al. (2022) [4]	The authors presented a patient with a history of hypertension, with chief complaints of chest pain, palpitations, and sweating for a one-day duration. In pain-free state upon arrival to the emergency department (ED). Normal cardiac enzyme	Case report	LCX, RCA, and diagonal 1	PCI
Mathew et al. (2022) [2]	The authors presented a patient with chest pain and hypertensive emergency with ECG finding significant for biphasic T waves V2-V5	Case report	Middle LAD and RCA	CABG
Obi et al. (2023) [25]	The authors presented a patient with atypical presentation of gastroparesis and uncontrolled type 2 diabetes with biphasic T waves V2-V5	Case report	Multivessel disease	CABG
Khattar et al. (2023) [5]	Multiple case presentation: Patient A is a 55-year-old male admitted for right carpal tunnel release; patient B is a 62-year-old female admitted for UTI. Both patients’ ECGs showed Wellens’ morphology. Both patients denied chest pain history and personal or family history of cardiovascular disease. Cardiac enzymes were within normal range. Both patients were previously diagnosed with COVID-19 over six months prior	Case report	LAD, RCA, and LCX	CABG
Wang et al. (2018) [6]	Multiple case presentation: Patient A is a 55-year-old female who presented to the ED with anginal chest pain. ECG showed Wellens’ morphology. Cardiac enzymes were within normal range. Patient B is a 85-year-old female who presented with paroxysmal chest pain and dyspnea. ECG showed Wellens’ syndrome	Case report	LAD and RCA	PCI
Jayasekara and Weerakoon (2016) [7]	Multiple case presentation: Patient A is a 58-year-old male who presented with intermittent central tightening chest pain radiating to the neck with diaphoresis for two days. He presents to the ED in pain-free state. ECG showed Wellens’ morphology. Cardiac enzymes were normal. Patient B was a 60-year-old male who complained of progressive episodic central constricting chest pain at rest with diaphoresis over one week	Case report	LAD, diagonal branch, and RCA	PCI and CABG
Parekh et al. (2022) [8]	The author presented a 48-year-old male with a past medical history of PTSD, ADHD, BPD unspecified, polysubstance use disorder, and hypertension who returned to the hospital for NSTEMI evaluation after he previously left against medical advice. The patient was pain-free on readmission. Urine drug screen was positive for cocaine, marijuana, and opiates	Case report	LAD, RCA, and LCX	CABG
Kim et al. (2020) [9]	An 81-year-old male with past medical history of moderate-severe aortic stenosis, hypertension, mild left ventricular hypertrophy, and hyperlipidemia presented to the emergency department with subacute-onset intermittent chest pressure at rest, decreased exercise tolerance, and occasional presyncope. The patient was pain-free on admission. ECG showed Wellens’ morphology. Cardiac enzymes were negative	Case report	LCX and RCA	PCI
Jaiswal and Boudreau (2016) [11]	A 59-year-old female with past medical history of familial hypercholesterolemia and hypertension and positive family history of myocardial infarction presented to the ED for left arm discomfort that developed overnight and resolved spontaneously. ECG showed Wellens’ morphology	Case report	LAD and LCX	PCI
Natarajan (2020) [12]	The authors presented a 67-year-old male who presented to the ED with chest pain for the last 12 hours. ECG showed Wellens’ morphology. Cardiac enzymes were within normal limits	Case report	LAD and LCX	PCI
Elkholy et al. (2021) [13]	An 86-year-old male with a past medical history of CAD, hypertension, diabetes mellitus (DM), hyperlipidemia, and possible chronic obstructive pulmonary disease presented to the ED with fever, vomiting, and dyspnea. COVID-19 diagnosis was confirmed. Initial ECG was unremarkable. Cardiac enzymes were trended upward, and repeat ECG showed Wellens’ morphology	Case report	RCA and LCX	PCI
Torelli et al. (2020) [15]	Patient A is a 75-year-old male with past medical history of DM, hypertension, and peripheral vascular disease who referred to the ED after an incidental finding of an altered ECG pattern during a pre-hospitalization visit. The patient presented in a pain-free state. ECG showed Wellens’ morphology. Visit for circumcision surgery. Patient B is a 57-year-old male patient with past medical history of hypertension and myocardial infarction with stent placement who presented to the ED due to altered ECG findings on a pre-hospitalization visit for a suppurative hidradenitis. The patient was pain-free on presentation. For both patients, cardiac enzymes were within normal range	Case report	LAD, LCX, and RCA	PCI
Di Stolfo et al. (2015) [16]	A 48-year-old male with past medical history of HIV presented for routine occupational evaluation. The patient was pain-free during the evaluation. He confirmed previous episode of anginal chest pain two months earlier. During that time, his cardiac enzymes and ECG were unremarkable. During his occupational evaluation, his ECG showed Wellens’ morphology	Case report	LAD	PCI
Mufti et al. (2018) [17]	An 87-year-old female with past medical history of hypertension, hyperlipidemia, paroxysmal atrial fibrillation, ischemic stroke without any residual deficits, and malignant melanoma status post resection and axillary lymph node dissection was admitted to the hospital for an elective skin grafting procedure by plastic surgery. Admission ECG showed a fib with left axis deviation, so she was admitted for monitoring. Subsequent ECG showed Wellens’ morphology. Cardiac enzymes were within normal limits. The patient denied chest pain	Case report	LAD	PCI
Yazdi and Sharim (2019) [18]	The authors presented a 70-year-old male patient who presented to the ED after an episode of chest pain the day prior that resolved after 10 minutes. ECG showed Wellens’ morphology. Cardiac enzymes were normal	Case report	LAD	No intervention
Yang et al. (2023) [19]	The authors presented a patient with past medical history of hypertension and T2DM who presented to the ED for paroxysmal chest pain. ECG showed sinus bradycardia with Wellens’ morphology	Case report	LAD	PCI
Plappert et al. (2022) [20]	A 46-year-old male presented to the ED for a three-day history of intermittent chest pain and diaphoresis that occurred at rest and resolved spontaneously. Cardiac enzymes were elevated	Case report	LAD	CABG
Tan et al. (2017) [21]	Patient A is a 61-year-old female with past medical history of HIV who presented to the ED with chest pain for eight hours. Initial ECG was unremarkable. Cardiac enzymes were mildly elevated. Repeat ECG showed Wellens’ morphology. The patient was pain-free at the time of repeat ECG. Patient B is a 49-year-old female with past medical history of HIV infection, T2DM, hypertension, systemic lupus erythematosus, and seizure disorder who presented to the ED for intermittent chest pain of one-day duration. Initial ECG was unremarkable, and cardiac enzymes were mildly elevated. Repeat ECG showed Wellens’ morphology	Case report	LAD	PCI
Khan et al. (2013) [22]	A 24-year-old female with past medical history of obesity, T2DM, and hypertension and family history of ischemic heart disease presented to the ED with a recent onset of chest pain. The patient had similar episode one week prior with unremarkable clinical workup. Pain-free ECG showed Wellens’ morphology. Cardiac enzymes were elevated	Case report	LAD	PCI
Shah et al. (2021) [23]	A 70-year-old male with past medical history of hypertension, hyperlipidemia, and GERD was presented to the ED for a five-day history of intermittent epigastric and chest pain. ECG showed Wellens’ morphology. Cardiac enzymes were within normal limits	Case report	LAD	Thrombectomy and PCI
Hollar et al. (2015) [24]	A 42-year-old male with family history of myocardial infarction presented to the ED for new onset of chest pain. ECG showed Wellens’ morphology	Case report	LAD	PCI

Discussion

In 1982, de Zwaan et al. discovered a unique type of proximal left anterior descending artery (LAD) T wave syndrome, which they named Wellens’ syndrome. This discovery was made while evaluating a subset of patients who had unstable angina during a stable and pain-free period [1]. Further investigation revealed that Wellens’ syndrome is linked to a substantial and critical stenosis of the LAD, and its presentation is unique, as it involves abnormal T wave changes in the precordial leads of patients suspected to have acute coronary syndrome. The electrocardiographic features of Wellens’ syndrome can be categorized into two distinct patterns: pattern A, which is characterized by biphasic T waves in V2-V3 (25% of cases), and pattern B, which shows symmetrical and deeply inverted T waves in the precordial chest leads (75% of cases) [3]. The diagnostic criteria for Wellens’ syndrome comprise several factors, including an ST segment elevation that is either isoelectric or minimally elevated (<1 mm), the absence of precordial Q waves, deeply inverted or biphasic T waves in V2-V3 that may extend to V1-V6, the presence of ECG pattern in a pain-free state, preserved precordial R wave progression, a recent history of angina, and normal or elevated serum cardiac markers [10].

This systemic review affirms that Wellens’ syndrome is an equivalent of ACS involving multivessel coronary arteries, with critical stenosis noted in the LAD, LCX, and RCA. This review indicated that although Wellens’ syndrome is seen mostly involving the LAD, it is not limited to it, as coronary artery blockages with Wellens’ syndrome pattern of ECG presentation that met diagnostic criteria can be seen in the LCX and RCA as part of a multi-coronary proximal artery occlusion. Most patients examined in this study had an unusual presentation of symptoms during a pain-free period, with the underlying pathophysiology linked to stenosis, reperfusion, and reocclusion of coronary vessels [4]. Despite the variability in the level of coronary artery involvement, this systematic analysis of selected primary research highlights the importance of early intervention, such as the utilization of an ECG followed by coronary artery angiogram (CAG), particularly in patients with significant risk factors for coronary artery disease upon presentation. Due to the absence of statistical information that would enable a meta-analysis of Wellens’ syndrome prevalence in coronary arteries other than the LAD, this systematic review followed a rigorous protocol in identifying primary research specifically related to the syndrome. The findings suggest that most cases examined had LAD involvement, supporting the hypothesis that LAD is the predominant location for the syndrome. However, the analyzed data also contributed to the investigation of whether Wellens’ syndrome is limited to the LAD, with reports showing that the diagnostic criteria are present in the LCX and RCA, as part of a multi-artery occlusion. This highlights the potential for bias in diagnosing the syndrome. This systematic review not only emphasizes that Wellens’ syndrome is a form of multivessel coronary artery disease that often presents atypically but also strongly advocates for early cardiac intervention in the form of PCI and/or CABG. The review suggests that medical management alone is insufficient in the treatment of Wellens’ syndrome.

## Conclusions

Our systemic review concluded that Wellens’ syndrome is a multivessel disease; although most prevalent in the LAD, it is also known to involve other coronary arteries such as the LCX and RCA or both. The importance of early recognition and treatment intervention is crucial, and as such, a patient with a typical presentation with coronary risk factor or a family history of a premature cardiac event should be evaluated with ECG, and if an ACS equivalent is noted or Wellens’ syndrome pattern is seen on ECG, urgent medical intervention is warranted.
